# De-intensification of adjuvant therapy in human papillomavirus-associated oropharyngeal cancer

**DOI:** 10.1186/s41199-016-0016-7

**Published:** 2016-12-20

**Authors:** Yi An, F. Christopher Holsinger, Zain A. Husain

**Affiliations:** 1 Department of Therapeutic Radiology, Yale University School of Medicine, Smilow Cancer Hospital LL 515, 35 Park St, New Haven, CT 06510 USA; 2 Division of Head and Neck Surgery, Department of Otolaryngology, Stanford University School of Medicine, 875 Blake Wilbur Drive, Palo Alto, CA 94305 USA

**Keywords:** Human Papilloma Virus (HPV), Oropharyngeal cancer, De-intensification, Adjuvant Therapy

## Abstract

Current adjuvant treatment guidelines for oropharyngeal squamous cell carcinoma treated with primary surgery are based on studies that predate the human papillomavirus (HPV) era. HPV-associated oropharynx carcinoma (HPV-OPC) has a much more favorable prognosis compared to HPV-unassociated cancer and is increasingly considered to be a distinct disease entity due to its unique etiology, presentation, and behavior. Currently, there is significant interest in adjuvant treatment de-intensification of HPV-OPC patients in order to reduce treatment-related toxicity while maintaining excellent clinical outcomes. Here, we review the evidence and rationale underlying the ongoing prospective trials of adjuvant treatment de-intensification for HPV-OPC patients.

## Background

The incidence of oropharyngeal cancer (OPC) has been increasing, in contrast to an overall decrease in all head and neck cancers rates [1, 2]. The rise in incidence of OPC has been attributed to the human-papillomavirus (HPV), and HPV-associated oropharyngeal carcinoma (HPV-OPC) is known to have distinct oncogenesis, clinical features, treatment response, and prognosis. Cigarette smoking is not thought to have a causative role in the development of HPV-OPC and this likely explains why its rising incidence differs from the overall trend of head and neck cancers [3]. High-risk HPV strains, predominantly HPV-16 and HPV-18, cause carcinogenesis via viral proteins E6 and E7, which inactivate tumor suppressors p53 and Rb, respectively. HPV-OPC is more likely found in younger males in developed countries with a limited smoking history and now comprises about 70% of all new oropharynx cancers in the United States [4]. Clinically, HPV-associated disease tends to present with smaller primary tumors, larger metastatic lymph nodes, and with histopathology more likely to have non-keratinizing, basaloid features and poor differentiation [5].

Evidence from secondary analyses of randomized clinical trials studying adjuvant treatments (RTOG 0234 and DKTK-ROG) have demonstrated superior clinical outcomes in HPV-associated patients treated with surgery and adjuvant chemoradiation [6–8]. For instance, HPV-OPC in RTOG 0234 had a significantly improved 2-years overall survival (OS) compared to the HPV-unassociated group (90.9% vs. 40% in cisplatin arm; 100% vs. 66.7% in docetaxel arm) [7]. Additionally, retrospective subgroup analyses of key prospective trials of definitive chemoradiation also show marked better performance of HPV-associated compared to HPV-unassociated cases (Table 1).

**Table 1:** Select prospective trials that demonstrated improved outcomes for HPV-associated disease on unplanned retrospective subgroup analysis

Trial	Comparison arms	Finding (HPV+ improved outcomes)
RTOG 0129 [44]	70 Gy over 7 weeks vs 72 Gy over 6 weeks. Both arms included concurrent cisplatin	8-years OS
		70.9% vs. 30.2%
RTOG 0522 [45]	Concurrent radiation with cisplatin +/- cetuximab	3-years OS
		85.6% vs. 60.1%
ECOG 2399 [46]	Phase II trial: 2 cycles of induction paclitaxel/carboplatin followed by radiation with concurrent paclitaxel	2-years OS
		95% vs. 62%
TROG 02.02 [47]	Radiation with concurrent cisplatin vs. radiation with concurrent cisplatin and tirapazamine	2-years OS
		91% vs. 74%
TAX 324 [48]	Induction docetaxel/cisplatin/fluorouracil followed by radiation with concurrent carboplatin vs. induction cisplatin/fluorouracil followed by radiation with concurrent carboplatin	5-years OS
		82% vs. 35%

Since HPV-OPC has a more favorable prognosis, treatment de-intensification for HPV-OPC has become a primary research objective, especially due to concern over long-term treatment toxicities. Long-term treatment-related morbidity may be even more impactful for HPV-OPC patients because of their potential for longer survival after therapy and younger age at diagnosis. Concerning treatment-related side effects include gastrostomy-tube requirement, with 1-year rates ranging from 4 to 18% after trans-oral surgery [9–11] and 5–10% after definitive chemoradiation with modern radiation techniques [12]. And even for patients who are not feeding tube dependent, dysphagia or aspiration can significantly decrease quality of life [13, 14].

## Main text

### Oropharynx carcinoma surgery

Standard management of OPC includes either frontline surgery or frontline radiation therapy, with or without concurrent chemotherapy. Frontline surgery includes not only resection of the primary tumor but also neck dissection with adjuvant therapy reserved for adverse pathologic features.

Primary surgery for OPC has experience a renaissance within the last decade [15]. Traditionally, primary surgery required an open trans-cervical approach with potential for considerable morbidity. As such, organ-preservation with definitive chemoradiation gained popularity in the 1990s and early 2000s. More recently, transoral endoscopic head and neck surgery (eHNS) has emerged as a functional organ preservation approach. eHNS consists of a minimally-invasive procedure using miniaturized instrumentation that avoids mandibulotomy and external incisions. eHNS is performed with carbon dioxide laser microsurgery or robotic surgical system and has been associated with a resurgence of primary surgical treatment for T1-T2 OPC [16]. Early reports have demonstrated that eHNS is associated with a lower complication rate and faster post-operative recovery [17, 18]. Moreover, oncologic outcomes from eHNS appear to be promising. A 2012 meta-analysis of over 500 OPSCC patients from 17 retrospective eHNS studies showed 1-year OS of over 90% and 2-year survival between 80 and 90% [18]. Most patients in this meta-analysis with standard indications for adjuvant treatment received adjuvant radiation or chemoradiation. Additionally, the meta-analysis found generally lower rates of functional deficits after trans-oral surgery, including a gastrostomy-tube dependence at 1-year of 0–9.5%. Aside from T1-2 disease, eHNS has also been used for locally-advanced OPSCC with acceptable clinical outcomes, suggesting upfront eHNS for selected stage III/IV OPC may also be appropriate [9, 19]. Recently, the largest multi-institutional report thus far of 410 head and neck cancer patients treated with eHNS, 89% of which were oropharyngeal primaries, found 2-year and 3-year loco-regional control rates of 91.8 and 88.8% with only 1 surgery-related mortality [20].

### Indications for adjuvant therapy

National Comprehensive Cancer Network (NCCN) guidelines recommend OPC patients after up-front surgery who have distinct adverse pathologic features receive either adjuvant radiation alone or chemoradiation [21]. Chemoradiation is recommended for high risk adverse features: extranodal extension (+ENE) and/or positive surgical margin(s). Other adverse pathologic factors are typically treated with adjuvant radiation alone. These are perhaps best summarized by the inclusion criteria of RTOG 0920, a phase III study of adjuvant radiation +/- cetuximab for head and neck squamous cell carcinoma (HNSCC) patients with intermediate risk factors. These intermediate risk factors include: close margin (<5 mm), ≥ 2 metastatic lymph nodes (LN) or a single LN > 3 cm, perineural invasion (PNI), lymphovascular invasion (LVI), T3 or T4a primary, or patients with initially focally positive margins but undergo re-excision with final negative margins. While not an inclusion criterion of RTOG 0920, nodal involvement of levels IV or V in oropharynx primary was also included by NCCN as an “intermediate” risk factor.

The evidence underlying the recommendation for chemoradiation for patients with high-risk features comes from two landmark studies, RTOG 9501 and EORTC 22931. In RTOG 9501, chemoradiation improved 2-years loco-regional control (82% vs. 72%, *p* = .01), and a disease-free survival benefit (reported HR 0.78), with the tradeoff of higher grade 3+ toxicity (77% vs. 34%) [22]. Conversely, EORTC 22931 demonstrated benefit of chemoradiation for local control (82% vs. 69%, *p* = .007), PFS (5-year estimate 47% vs. 36%), and 5-years OS (53% vs. 40%, *p* = .02) [23]. The two studies had notably slightly different inclusion criteria (Table 2). In RTOG 9501, high-risk was defined as 2 or more metastatic LN, +ENE, or positive surgical margins [24]. Inclusion criteria of EORTC 22931 were more broad and included pT3-4 N0-3, pT1-2 N0-1 with + ENE, positive surgical margins, PNI, or LVI, and oral cavity or oropharyngeal primary cancers with level IV or V lymph nodes (additional criteria in Table 2). In order to make sense of who benefits most from chemotherapy, a subsequent combined analysis of the EORTC 22931 and RTOG 9501 trials and subsequently found + ENE and positive margins were the only features that statistically predicted overall survival benefit with chemoradiation [25].

**Table 2:** Inclusion criteria for select randomized adjuvant treatment trials comparing chemoradiation versus radiation alone

Trial	Inclusion criteria
EORTC 22931 [23]	Sites: oral cavity, oropharynx, larynx, or hypopharynx
	1 of the following:
	• pT3-4 N0-3 except for T3N0 of the larynx with negative resection margins • pT1-2 N2-3 • pT1-2 N0-1 and positive margins, PNI, or vascular embolism (lymphovascular invasion) • oral cavity or oropharyngeal primaries with level IV or V lymph node involvement
RTOG 9501 [22]	Sites: oral cavity, oropharynx, larynx, or hypopharynx Status post complete macroscopic resection
	1 of the following
	• two or more metastatic regional lymph nodes • extracapsular extension • microscopically involved mucosal margins of resection

### The prognosis of extranodal extension in HPV-OPC

Given the favorable prognosis of HPV-OPC, the question has been raised whether ENE carries the same adverse prognostic value in this favorable group of patients. Several concerns arise with applying findings from RTOG 9501 and EORTC 22931 to HPV-OPC patients. HPV status testing was not routinely performed in the two trials. Furthermore, oropharyngeal primaries made up only 42 and 30% of the RTOG 9501 and ECOG 22931, respectively, suggesting that HPV-OPC made up a minority of patients in those studies.

Several other issues arise when trying to understand the prognostic implications of ENE amongst HPV-OPC patients, not the least of which is there may be significant intra- and inter-observer variability in assessing ENE. In a study from Washington University with 152 patients, slides were re-examined by a single study-pathologist and the results were compared to the original pathology report. ENE was reported to be present in 124/152 cases or 82% of cases as read on the initial pathology reports but only present in 79/152, or only 52%, on re-review [26]. It is troubling that even within a single high-volume center there can be a thirty percent discrepancy in pathologic assessment of ENE. In a later study, the same authors created digitally scanned nodal metastases slides, which were initially read by five different pathologists. These pathologists were instructed to perform a dichotomous assessment for ENE as either present or absent, and there was agreement in only 48% of cases. Subsequently, the pathologists were given a defined system which grades ENE into four categories, and agreement improved slightly to 64% [27].

This study, in turn, raises another point: while the seminal studies describing the benefit of chemoradiation in ENE positive patients graded ENE dichotomously as present or absent, more recent investigation now suggests the extent of ENE may be better characterized as a spectrum, and the degree of ENE may matter as well. In the aforementioned Washington University experience, the pathology reporting of the presence or absence of ENE did not correlate with disease-free survival (DFS). However, when ENE was graded on the four-point system, the group with grade 4, defined by the presence of soft tissue metastasis (STM), had a reduced DFS compared to patients without STM (80% vs 93%, *p* = .02) (see Table 3 for their ENE grading system). Taken together, this suggests that extent of ENE is important to consider, and not all patients with ENE may have a similar prognosis. This approach has been adopted in the ECOG 3311 trial, where patients with ≤ 1 mm of ENE are enrolled in the radiation alone arm. For patients with >1 mm, post-operative chemoradiation is given in this high-risk arm.

**Table 3:** Grading system for extranodal extension for metastatic lymph node established by Lewis *et al* [49].

Extra Nodal Extension Grading System		
Grade	Histologic Finding	ENE positivity
0	Tumor is surrounded by lymphoid tissue	–
1	Tumor reaches the capsule (with no intervening lymphoid tissue) with thickening of overlying capsule	–
2	Tumor in perinodal tissue, extending ≤ 1 mm beyond capsule	+
3	Tumor in perinodal tissue and extending > 1 mm beyond capsule	+
4	Tumor mass without residual nodal tissue or architecture. No residual lymphoid architecture or germinal center.	+

Higher grader corresponds with more severe ENE. Grades 0–1 are usually defined as negative ENE while grades 2–4 are defined as positive ENE. Grade 4 also designated as *Soft Tissue Metastasis*

Studies have also examined whether ENE in patients with HPV-OPC is a risk factor for disease-free survival following surgical resection. In the Washington University experience of p16+ OPC patients after eHNS, the rate of DFS was not significantly different amongst patients whose pathology reports described ENE versus those without, with 3-year disease-specific survival (DFS) estimates of 89% (95% CI 84–95%) and 94% (95% CI 83–100%), respectively [26]. These results were corroborated by a study from the University of Pittsburgh that examined 76 patients with HPV-OPC, 45 of which had ENE. There was no difference in rates of DSS amongst patients with or without ENE (*p* = 0.936) [28]. In a subsequent update, these patients were reported to have a 5-year DSS of 84.8% (95% CI 64.4–94.05%) in patients with ENE and 89.3% (95% CI 73.9–98.1%) in patient without ENE [29].

The most provocative evidence suggesting a lack of benefit from concurrent chemoradiation for patients with HPV-OPC comes from retrospective comparisons of patients with ENE that received either radiation therapy alone or chemoradiation. In the Washington University series, 113 patients had ENE graded on their pathology reports, and, of this group, 48 were treated with radiation alone while 65 were treated with chemoradiation. The 3-year DFS rates were similar at 94.5% in the radiation group and 91.8% in the chemoradiation group (*p* = .74) [26]. These values should of course be taken with the caveats associated with a single institution retrospective study, and the results could of course be reflective of patient selection, but nonetheless it remains a notable finding.

Taken together, the benefit of chemoradiation in HPV-OPC patients with ENE remains a pivotal unanswered question in modern head and neck oncology. The underlying recommendation for chemoradiation for ENE positive patients came from a larger group of HNSCC most of which were likely not HPV-associated, and therefore carry a different biology, natural history, and a significantly worse prognosis. In addition, the historic dichotomization of ENE may actually be an oversimplification of the true risk of recurrence, and further efforts are needed to standardize the reporting of ENE across centers. The current data, limited to small single institution series at high volume centers, shows that ENE does not appear to be necessarily associated with worse disease specific survival, and that patients who received radiation seemed to do similarly to those who receive chemoradiation. Given the additional toxicity with chemoradiation, which is associated with more than double the rate of grade 3 or higher acute toxicity in comparison to radiation alone [22], answering the question of whether concurrent chemotherapy is necessary for patients with ENE is a critical priority for the head and neck oncology community.

#### Number of positive lymph nodes

Attention has recently been paid to whether patients with a higher number of pathologically involved lymph nodes represent a separate high-risk group. A study of HPV-OPC after eHNS from Washington University found the presence of five or more involved LN was associated with a recurrence rate of 24% on multivariable analysis (OR 3.12) [30]. Additionally, a recent study from the SEER database demonstrated that oropharyngeal carcinoma patients with 5 or more involved lymph nodes had a worsened survival, although this study was limited in that information on HPV and p16 testing was unavailable [31]. In contrast, the recently proposed staging system for HPV-OPC, the ICON-S group concluded that the number of positive lymph nodes was not predictive of OS, though this was in a group of patients that largely had clinically as opposed to pathologically staged nodal disease [32]. ECOG 3311 includes patients with five or more involved nodes in its high-risk group to receive adjuvant chemoradiation, and results from this study should provide needed prospective data about nodal number and the risks of recurrence [33].

### Post-operative radiation dose

There is considerable variation among studies in terms of radiation dose used in the adjuvant setting, and whether this should be affected by high risk factors such as ENE or positive margins. Fortunately, there is randomized data to help provide guidance. Peters et al. performed a Phase III study investigating the optimal adjuvant radiation dose required based on postoperative risk stratification. The study included 240 patients, and nearly all were stage III/IV oral cavity, oropharynx, hypopharynx, or larynx primaries after initial resection. Patients were assigned into low-risk or high-risk groups for both primary site and nodal disease based on a point system incorporating T-stage, margins status, and nerve involvement for primary site and nodal number, and nodal groups for nodal disease. Low risk patients were randomized to a dose of 57.6 Gy (initially < 54 Gy) vs. 63 Gy, and high-risk patients were randomized to 68.4 Gy vs. 63 Gy. The 2-years loco-regional control rate for the low risk primary site group was 92% for 57.6 Gy, 89% for 63 Gy, but only 63% for <54 Gy. This led the authors to conclude 57.6 Gy in 1.8 Gy fractions was the recommended dose for postoperative patients with intermediate risk factors. In the high-risk group, 2-year local control was 89% with 63 Gy and 81% with 68.4 Gy. There was a noticeable local control dose-response in the subgroup of ENE+ cases, as among ENE+ cases, the 2-years control rate was 52% for 57.6 Gy, 74% for 63 Gy, and 72% for 68.4 Gy [34]. With these findings, the recommended postoperative radiation dose for ENE+ patients was 63 Gy in 1.8 Gy fractions. Interestingly a similar dose response was not seen for patients with positive margins. Despite this level 1 evidence, these doses were never widely adopted. Practitioners have largely moved to using a daily dose of at least 2 Gy per day, given data that showed a benefit to shorter treatment courses [35, 36]. Using the linear quadratic equation, 57.6 Gy in 1.8 Gy fractions translates to approximately 56–58 Gy in 2 Gy fractions, and 63 Gy in 1.8 Gy fractions translates to approximately 62 Gy in 2 Gy fractions. Perhaps for reasons more logistical than scientific, this has often translated to a dose of 60 Gy to areas of potential microscopic disease, with some pushing for a boost to areas of ENE or positive margins to 66 Gy. This variation is seen even among large cooperative group trials. Current studies of intermediate risk patients, such as RTOG 0920, and the standard arm of ECOG 3311 use a dose of 60Gy. For high-risk patients, RTOG 9501 mainly used 60 Gy though 13% received an optional 6 Gy boost while EORTC 22931 mainly used 66 Gy. The current high-risk study for p16(-) cancers, RTOG 1216, calls for 60 Gy with an optional 6 Gy boost given concurrently. ECOG 3311, which is limited to p16 positive patients, uses 66 Gy in its high-risk arm. This variation is reflected in national practice guidelines which call for a dose to high-risk sites (positive margins and/or + ENE) of 60–66 Gy in 2 Gy fractions and a dose to intermediate risk sites of 54–63 Gy using simultaneous dosing techniques [21]. Recent evidence, however, suggests a lack of benefit for higher doses in HPV-associated oropharyngeal cancer. Investigators at Washington University compared outcomes for patients with HPV-associated OPSCC and the risk factors of close or positive margins and/or ECE, but treated to different radiation doses [37]. The institutional standard dose for such patients was 66 Gy from 1998 to 2009 but was changed to 60 Gy in 2009. Researchers found no significant differences in 2-year loco-regional recurrence-free survival, cause-specific survival or overall survival between 60 Gy and 66 Gy groups, suggesting 60Gy may be a reasonable adjuvant radiation dose in HPV-associated OPSCC.

### Omitting adjuvant radiation to the primary tumor bed

Another potential de-intensification approach is consideration of omitting adjuvant radiation to the primary tumor bed. The rationale here is that given the improved margin control of eHNS and low rates of tumor bed recurrence seen in early stage patients who are observed after eHNS, primary site irradiation may not be necessary. The indication for irradiation in many early stage patients ends up being the presence of multiple involved lymph nodes, and thus it is hypothesized that perhaps, the neck alone can be irradiated, translating to smaller radiation volumes and potentially less morbidity.

A recently published study of p16+ oropharyngeal SCC patients after eHNS found a local recurrence rate of only 3% (3/92) in pT1-T2 patients who did not receive adjuvant radiation to the primary tumor bed, although approximately half did receive neck directed irradiation [38]. Those spared adjuvant RT to the primary bed were also found to have lower gastrostomy tube rates (10% vs 2% 1-year rate for pT1-T2). The authors concluded their findings suggest that omitting primary tumor adjuvant radiation in low-risk, early T-stage, HPV-associated oropharyngeal carcinoma patients appears safe and may be associated with less functional morbidity.

Of note, while in theory eliminating the primary tumor bed as a target would theoretically lead to a substantial decrease in radiation to normal tissues, dosimetric studies comparing primary plus neck irradiation with neck-only irradiation suggest only a modest potential benefit with this approach [39, 40]. This may be due to the fact that the level II lymph nodes, which represent the first echelon nodal drainage for oropharyngeal cancers, and are thus always in the neck radiation volume, sit adjacent to the tonsil and base of tongue. An dosimetric analysis of radiation for tonsillar cancer patients compared tonsil/post-operative bed doses for patients treated to the unilateral neck only, and found that the primary site still received a mean dose of 53.9 Gy [39]. Additionally, the radiation dose to bilateral parotid glands, larynx or mandible was not significantly lower in the neck-only radiation treatment plans. There was however a slight benefit in dose to the oral cavity (34.0 Gy vs. 29.8 Gy *p* = 0.002) and superior pharyngeal constrictors (46.1 Gy vs. 42.9 Gy *p* = 0.01). A separate study of base of tongue tumors compared radiation plans of patients receiving adjuvant radiation to the bilateral neck and primary tumor to those same patients replanned to receive only bilateral neck radiation and found a decrease in oral cavity dose (47.4 Gy vs. 22.3 Gy) but no significant benefit for contralateral parotid or pharyngeal constrictor muscles [40]. Of note, the primary tumor bed still received a mean dose of 40.2 Gy, which could complicate consideration of future re-irradiation for any patients who may recur.

Currently, a University of Pennsylvania phase-II trial is underway that will further elucidate the benefits of neck only irradiation in HPV-associated OPSCC patients after eHNS (NCT02159703). The ADEPT adjuvant trial will also allow omission of primary bed radiation in pT1-2 patients with a negative surgical margin who still have an indication for neck irradiation (NCT01687413).

### Current treatment de-intensification trials with primary surgery in HPV-OPC

Several ongoing trials are analyzing treatment de-intensification in the adjuvant setting, and are summarized in Table 4. Below is a discussion of some of the larger trials. ECOG 3311 stratifies patients into three risk groups. The low risk group requires negative margin, 0–1 involved LN, and negative ENE, and do not receive any adjuvant therapy. The intermediate risk group, which includes negative margin but ≤1 mm ENE or 2–4 involved LN, is randomized to receive either standard adjuvant radiation of 60 Gy or de-escalated dose of 50 Gy. High-risk patients, with either positive margin, >1 mm or ≥ 4 involved LN, invariably receive 66 Gy with concurrent cisplatin, which is in line with standard treatment recommendations.

**Table 4:** Active Phase II/III Trials of Surgically-treated HPV-OPC

Trial	Details	Inclusion Criteria	Stratify	Definition	Treatment
ECOG 3311 [33]	Phase II RCT Multi-site	Post trans-oral surgery Stage III, IVA, IVB	Low	R0 0–1 LN+ -ECE	Observation
			Intermediate	R0 2–4 LN+ +ENE (≤1 mm)	*Randomized*: 50 in 25 fx Gy vs 60 Gy in 30 fx
			High	R1-2 5+ LN+ +ENE (>1 mm)	66 Gy with weekly cisplatin (40 mg/m^2^ weekly)
ADEPT [50]	Phase III RCT WashU	Post trans-oral surgery Stage III, IV +ENE, R0	None		*Randomized*: 60 Gy +/- cisplatin (40 mg/m^2^ weekly)
PATHOS [51]	Phase II/III UK, multi-site T1–T3 N0-N2b	Post trans-oral surgery T1-3, N1-2b	Low	T1-T2	Observation
			Intermediate	T3 T1-T2 with: • N2a/2b • PNI • LVI • Close margins (1–5 mm)	*Randomized*: 50 Gy in 25 fx vs 60 Gy in 30 fx
			High	Positive margin (<1 mm) Negative marginal biopsy +ENE	*Randomized*: 60 Gy +/- cisplatin (40 mg/m^2^ weekly)
SIRS [52]	Phase II Mount Sinal	Post-surgery T1N1-2b or T2N0-2b	Low	R0 -LVI, -PNI, no LN+	observation
			Intermediate	R0 +LVI +PNI <3 LN+ ENE (<1 mm)	50 Gy
			High	R1/R2 3+ LN+ ENE (≥1 mm) Matted or SCV nodes	56 Gy with cisplatin (40 mg/m^2^ weekly)
Mayo Clinic [41]	Phase II	Post-surgery Stage I-IVB R0 resection	At least one of following: • LN > 3 cm • >1 LN+ • PNI • LVI • T3 or microscopic T4a • +ENE		Concurrent chemoradiation: Accelerated/hyperfractionated: 36 Gy in 20 fractions • BID 5 days/week, days 1-12 • Concurrent docetaxel
University of Pennsylvania [43]	Phase II	Post-TORS and Selective Neck Dissection pT1-T2, N2a-c	Notable exclusion criteria: • Positive or microscopically positive surgical margins (negative or close (<2 mm) margins of primary cancer • PNI of primary cancer		All trial patients forgo adjuvant radiotherapy to the primary cancer

*R0* negative surgical margin, *R1/R2* positive surgical margin, *PNI* perineural invasion, *LVI* lymphovascular invasion, *ENE* extranodal extension, *LN+* involved lymph nodes, *SCV nodes* supraclavicular lymph nodes

The PATHOS trial, funded by Cancer Research UK, is similar to ECOG 3311 since it also calls for post-eHNS stratification into low, intermediate, and high-risk groups. However, the PATHOS trial employs different criteria for intermediate and high-risk groups. Intermediate-risk inclusion criteria are: negative margin, at least pT3 disease or pT1-2 disease with pN2a/b, PNI, LVI, or close margin (defined as 1–5 mm). Intermediate risk patients are randomized to standard arm of 60 Gy in 30 fractions or dose-de-escalated to 50 Gy in 25 fractions. The high-risk group requires positive margins or + ENE, and these patients are randomized to 60 Gy with cisplatin or without concurrent chemotherapy.

Similar to the PATHOS high-risk arm, the ADEPT phase III trial will also elucidate if adjuvant chemotherapy is needed for HPV-OPC patients with + ENE. Closed to accrual, the ADEPT trial includes post-eHNS patients with + ENE but negative surgical margins. Patients are randomized to the standard 60 Gy with concurrent cisplatin or de-intensified to 60 Gy without chemotherapy.

Additionally, a single-arm Mayo Clinic phase II trial underway is studying the use of an accelerated hyperfractionated twice daily (BID) radiation in post-operative cases [41]. The radiation schedule consists of 36 Gy in 20 fractions in 1.8 Gy BID dosing and delivered 5 days a week within the first 12 days with concurrent docetaxel. This reduced radiation dosing is inspired by studies showing lower doses were able to control disease in anal cancer, another HPV-associated squamous cell carcinoma [42]. Eligible patients must have at least one of the following risk factors: LN > 3 cm, two or more metastatic LN, perineural invasion, LVSI, pT3, or microscopic pT4a stage.

Finally, there is also an ongoing University of Pennsylvania single-arm phase II trial of pT1-T2, N2a-c patients after eHNS who receive adjuvant radiation only to the neck and not the primary tumor bed [43]. This study addresses the question can the primary tumor bed forgo adjuvant radiation and potentially reduce toxicity if the only indication for adjuvant irradiation is multiple involved neck nodes (without risk factors at the primary site) [43].

## Conclusions

The superior prognosis of HPV-OPC has catalyzed clinical investigation of treatment de-intensification. Ongoing phase II and phase III trials will provide novel risk-adjusted adjuvant treatment schemes and provide answers regarding the appropriateness of radiation dose reduction or reduction of systemic therapy in HPV-OPC. These studies will also answer if traditional adverse post-operative risk factors, such as + ENE or positive margins, have the same prognostic value as in HPV-associated cohort. It should be noted, however, that therapeutic de-escalation is a hypothesis requiring clinical study - at this time HPV-OPC patients should continue to receive standard of care adjuvant treatment according to national guidelines unless on a clinical trial.

## Abbreviations

CR: complete response
DFS: disease-free survival
eHNS: transoral endoscopic head and neck surgery
ENE: extranodal extension
HNSCC: head and neck squamous cell carcinoma
HPV: human papillomavirus
HPV-OPC: HPV-associated oropharynx carcinoma
LN: metastatic lymph nodes
LVI: lymphovascular invasion
OPC: oropharyngeal cancer
PNI: perineural invasion
STM: soft tissue metastasis
